# Retrospective Studies with Small Sample Size are Still Dominating Colon Cancer Research: Local Recurrence in Right Sided Colon Cancer as a Case in Point

**DOI:** 10.5152/tjg.2025.24749

**Published:** 2025-03-19

**Authors:** Khan Yusra, Zhi Sean Teng, Sayyed Raza, Alaa El-Hussuna

**Affiliations:** 1 Department of General Surgery, Patel Hospital, Karachi, Pakistan; 2 Universiti Malaya Faculty of Medicine, Kuala Lumpur, Malaysia; 3 The Royal Marsden Hospital, London, United Kingdom; 4 OpenSourceResearch organisation (OSRC.network), Alborg, Denmark

## Introduction

Worldwide, over US$100 billion is invested every year in supporting biomedical research, resulting in estimated 1 million research publications per year.^1^ This increased investment in research, irrespective of the sponsors, means more attention must be paid to research quality if resources are to be translated into knowledge that advances patient care. Re-producing the existing evidence is very tempting and may explain why unnecessarily or poorly designed research is produced.

The aim of this study was to examine dominant study designs and investigate redundancy in surgical research.

## Method

The authors conducted a systematic review according to a pre-defined protocol published on PROSPERO (an international database of prospectively registered systematic reviews in health ID=CRD42021231233).^2^ The review was conducted according to the recommendations in the Cochrane Handbook for Reviews of Interventions and was reported according to the Preferred Reporting Items for Systematic Reviews and Meta-Analyses (PRISMA) statement.

Peer-reviewed studies that reported local—regional recurrence or survival rates after right-sided curative colon resection were included. Publication years were limited to 2010-2021. Two authors (Y.K., Z.S.T.) independently screened titles and abstracts for eligibility, while another author (A.E.) reviewed all the included abstracts to ensure they meet inclusion criteria.

## Results

**Figure 1.**
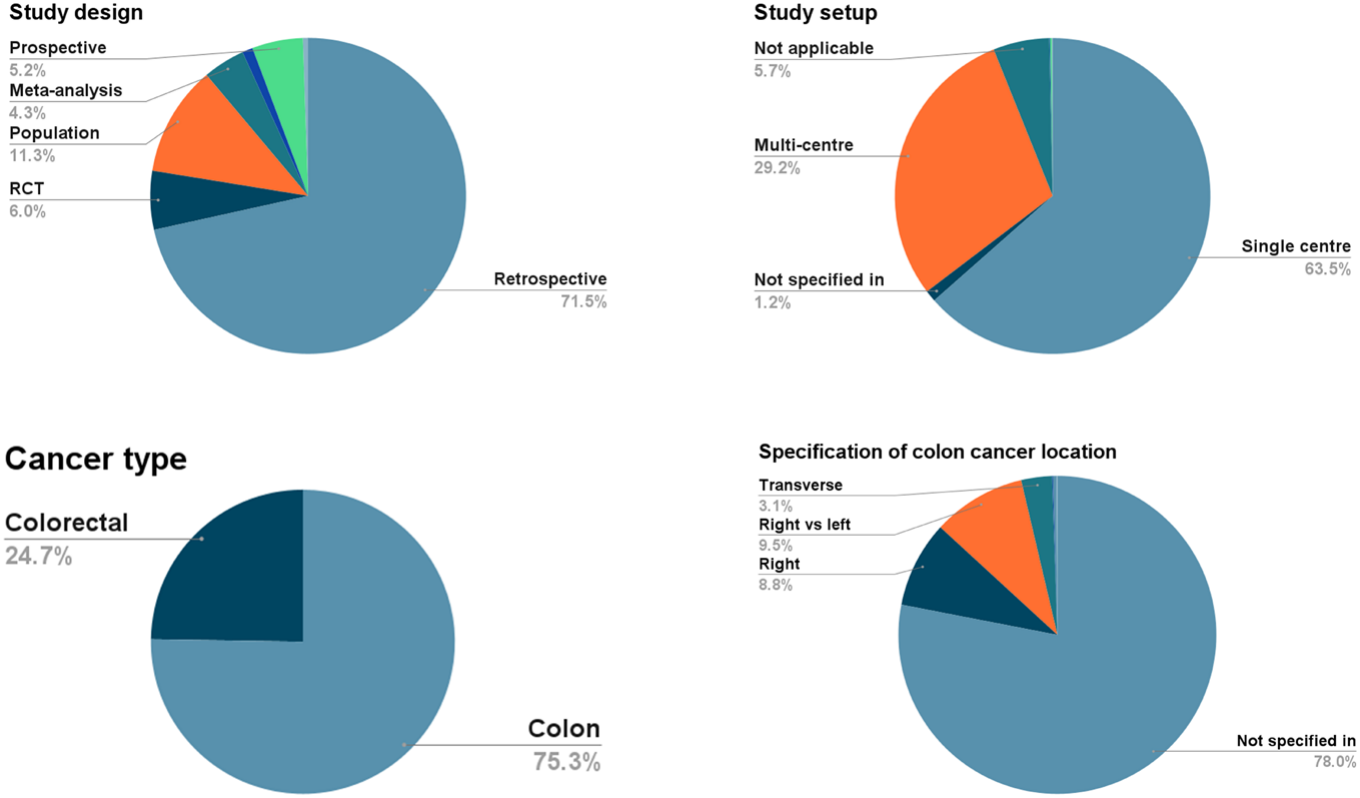
Types of study designs in 1116 peer-reviewed studies included in the systematic review after the exclusion of conference abstracts. The study set-up shows how different studies reported results about cancer location.

About 17 456 articles were identified on searching PubMed.gov, Embase, and Google Scholar. After removing duplicates, 1633 studies’ abstracts were included. Published conference abstracts were excluded, leaving 1116 peer-reviewed study abstracts. The study design is shown in Figure 1.

**Figure 2.**
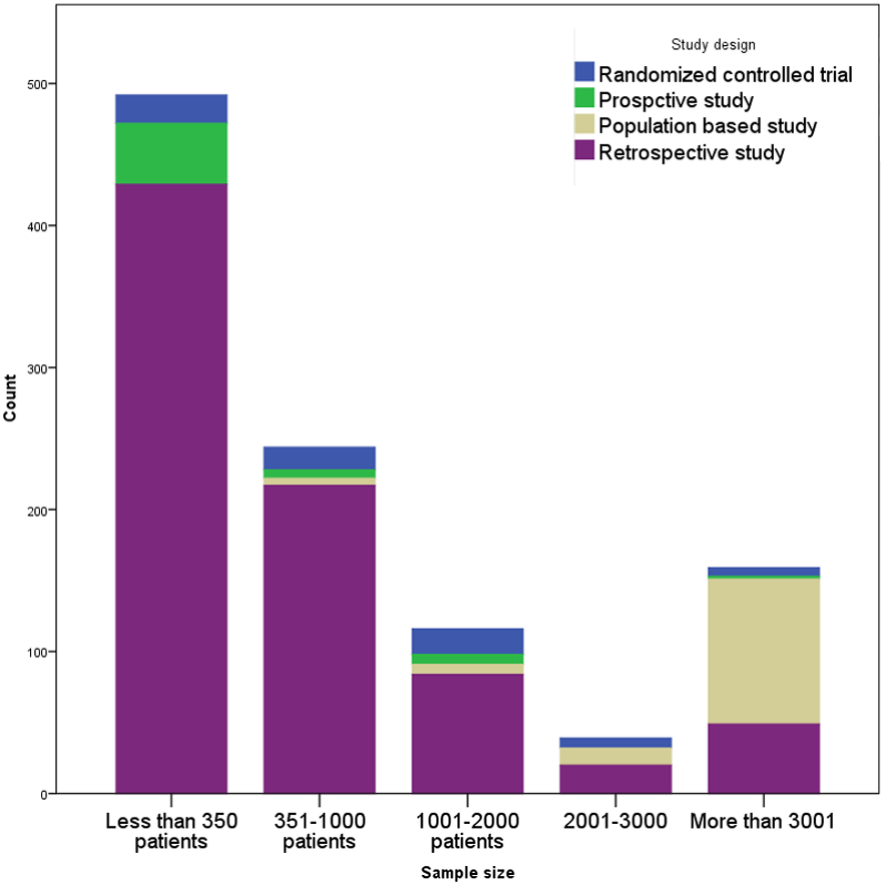
Sample size in the 4 study types. Assuming an adequate sample size of 350 patients, then 60% of retrospective studies, 75% of prospective studies, and 50% of RCTs were underpowered.

Figure 2 shows that assuming an adequate sample size is 350 patients,^3^ then 60% of the retrospective studies and 75% of the prospective studies were underpowered while 50% of the Randomized Controlled Trials (RCTs) satisfied this condition. The retrospective studies were conducted in a single center (82%), 77% did not specify the location of cancer, and 25% investigated mixed population of colorectal cancers. This trend was somewhat better in the prospective studies, with 30% conducted in a multi-center setup and were more specifically designed to investigate colon cancer (80%).

RCT sample sizes ranged from 60 to 6088 patients. Eighty-two percent were multi-center studies designed specifically to address colon cancer, although only 10% specified the tumor location in the colon. Seventy-one percent of the RCTs were conducted by oncologists, while surgeons contributed to 26%. The surgical RCTs examined access to the abdominal cavity (laparoscopic vs. open), mechanical bowel preparation, and surveillance after resection. All these RCTs were conducted in developed countries, with Japan leading (30%) followed by the United States (18%). By region, most studies were conducted in the Western Pacific Region (45%) followed by the European Region (32%).

## Discussion

The systematic review focused on right-sided colon cancer underscores a critical aspect of current research practices—predominantly, the prevalence of single-center studies and their underpowered nature. This prevalent issue underscores a deeper challenge within surgical research, created by studies with similar focus areas without significantly advancing the field.^4^ Furthermore, the notable disparity between the designs of retrospective and prospective studies, and their geographical concentration, sheds light on the need for a more diversified and comprehensive approach in conducting surgical research.

The study proposes an imperative to pivot towards larger, international, multicenter studies. Such a transition is not just about increasing the number or scale of studies but about enhancing the quality and applicability of research outcomes. The emphasis on the need for studies to specify the tumor location within colon cancer research further indicates a gap in current research practices, potentially limiting the applicability and relevance of study findings to clinical practice.

In conclusion, this study sets forth a clear call to action for the research community.^5^ By advocating for a strategic shift towards well-designed, larger, and more detailed studies, it paves the way for meaningful advancements in the field.

## Data Availability

The data that support the findings of this study are available on request from the corresponding author.
